# T-RHEX-RNAseq – a tagmentation-based, rRNA blocked, random hexamer primed RNAseq method for generating stranded RNAseq libraries directly from very low numbers of lysed cells

**DOI:** 10.1186/s12864-023-09279-4

**Published:** 2023-04-17

**Authors:** Charlotte Gustafsson, Julia Hauenstein, Nicolai Frengen, Aleksandra Krstic, Sidinh Luc, Robert Månsson

**Affiliations:** 1 Department of Laboratory Medicine, Division of Clinical Immunology, Karolinska Institutet, ANA Futura, Alfred Nobels Allé 8 floor 7, Huddinge, SE-141 52 Sweden; 2 Department of Clinical Genetics, Karolinska University Hospital, Stockholm, Sweden; 3 Center for Hematology and Regenerative Medicine (HERM), Karolinska Institutet, Stockholm, Sweden; 4 Department of Medicine Huddinge, Karolinska Institutet, Stockholm, Sweden; 5 Department of Hematology, Karolinska University Hospital, Stockholm, Sweden; 6 Department of Clinical Immunology and Transfusion Medicine, Karolinska University Hospital, Stockholm, Sweden

**Keywords:** Stranded RNAseq, Tagmentation, Random hexamer priming, RNA purification free, Expression profiling

## Abstract

**Background:**

RNA sequencing has become the mainstay for studies of gene expression. Still, analysis of rare cells with random hexamer priming – to allow analysis of a broader range of transcripts – remains challenging.

**Results:**

We here describe a tagmentation-based, rRNA blocked, random hexamer primed RNAseq approach (T-RHEX-RNAseq) for generating stranded RNAseq libraries from very low numbers of FACS sorted cells without RNA purification steps.

**Conclusion:**

T-RHEX-RNAseq provides an easy-to-use, time efficient and automation compatible method for generating stranded RNAseq libraries from rare cells.

**Supplementary Information:**

The online version contains supplementary material available at 10.1186/s12864-023-09279-4.

## Background

The field of transcriptomics has during the last two decades evolved from analysis of select genes with PCR-based methods, to broad analysis using microarrays and finally, to the now standard use of RNA sequencing (RNAseq) to characterize full transcriptomes [1]. Principally, RNAseq is achieved by reverse transcribing RNA into cDNA, introducing sequencing adapters and applying next-generation sequencing to determine the presence of transcripts.

Through this process, the strand-of-origin information can be maintained which allows for accurately resolving sense and antisense transcription [2, 3]. A common strategy is the incorporation of deoxy-UTP (dUTP) during second strand cDNA synthesis [4], followed by a digestion of the uracil containing strand before amplification of the library.

The sequencing of full length polyadenylated RNA – using oligo-dT priming – has been the mainstay for RNAseq. However, better understanding of RNA biology has come with increased interest in studying other RNA species or incompletely processed RNA lacking polyadenylation [1]. The use of random hexamer priming allows for analyzing a broader range of RNA species, but comes with the drawback that ribosomal RNA (rRNA) – that can constitute up to 90% of total RNA – is reverse transcribed together with the RNA of interest. This makes a reduction of rRNA representation in the library an attractive strategy for limiting the need for extensive sequencing. Several strategies to restrict inclusion of rRNA have been developed, including depletion of rRNA sequences [5–7], enzymatic degradation of rRNA [8, 9] or simply blocking of rRNA from undergoing reverse transcription [10].

Early RNAseq libraries were generated by ligation of sequencing adapters to double stranded (ds) cDNA in a multi-step process. The use of transposase (Tn5) to fragment ds DNA and integrate sequencing adapters in a single step – so called tagmentation – was first described by Adey et al., [11]. Because of the simplicity, tagmentation has seen wide use in genomics applications ranging from whole genome sequencing [12] to ATACseq [13] and ChIPseq [14, 15]. In collaboration with Epicenter, Gertz et al., [16] published the Directional Tn-RNAseq method and demonstrated that tagmentation in combination with dUTP incorporation could be used to generate stranded RNAseq libraries. In a refined version, this method was briefly made commercially available from Epicenter as the TotalScript kit. The kit provided a straightforward procedure for generating random hexamer primed stranded RNAseq libraries from 1-5 ng of total RNA, although an effective solution for reducing the rRNA contribution to the library was lacking. Analogous to how stranded RNAseq libraries were generated using forked adapters and dUTP incorporation [4], the Directional Tn-RNAseq method relied on introducing the i5 adapter on the 5’ side of the transposed cDNA. This was achieved by introducing only the i5 adapter in the tagmentation step. The i7 adapter was subsequently introduced by replacing the part of the i5 adapter that is not covalently linked to the tagmented DNA with an i7 adapter oligo. Noteworthily, this allows for sequencing of all tagmented molecules, in contrast to standard tagmentation where only half the library fragments acquire the i5-i7 combination needed for sequencing. With the PCR amplification of the library being performed after the tagmentation step, the protocol further allowed for using the recurrence of the same Tn5 integration sites to estimate and compare the potential contribution of biased PCR amplification to the library.

The current market offers several commercial kits for generating RNAseq libraries from ≤ 1 ng of RNA, including the SMARTer Stranded total RNA-seq Pico kits (Takara), NEBNext Single cell/Low input RNA prep kit (New England Biolabs; NEB), and Ovation SoLo RNAseq preparation kit (Tecan). However, none of these offers a solution with the combinatorial use of cell lysates as input for the cDNA synthesis, random hexamer priming, and effective removal of rRNA, while at the same time taking advantage of the streamlined workflows that can be achieved using tagmentation for RNAseq [17–20]. Moreover, a multitude of RNAseq methods have been developed for the analysis of single cells, including SuperSeq [21], MATQseq [22], RamdaSeq [23], SMARTseqTOTAL [24], Smart-Seq3xpress [25] and VASA-seq [26]. Out of these, only VASA-seq allows for strand-specific analysis of transcripts regardless of poly-adenylation. However, the elegant single cell adaptations of the VASA-seq protocol also make it a complex procedure for performing RNAseq on bulk cells.

To establish a convenient RNAseq protocol for analyzing a wide spectrum of RNA in low numbers of FACS sorted cells, we revisited the use of tagmentation with one adapter to achieve stranded RNAseq libraries [16]. Based on the re-creation and refinement of this approach, we here present a tagmentation-based, rRNA blocked, random hexamer primed RNAseq method (T-RHEX-RNAseq) for generating stranded RNAseq libraries from cells without prior RNA purification. T-RHEX-RNAseq is technically straightforward, time efficient, compatible with automation and can be performed on input material ranging from nanograms of purified total RNA down to less than a hundred FACS sorted cells.

## Results

### Re-establishing directional tagmentation-based RNAseq library construction

The Directional Tn-RNAseq protocol by Gertz et al. [16], in brief relied on synthesis of ds cDNA with dUTP incorporation in the second strand, tagmentation with i5 compatible adapters alone, introduction of the i7 adapter by oligo replacement, digestion of the dUTP containing strand and amplification of the library followed by sequencing.

**Fig. 1:**
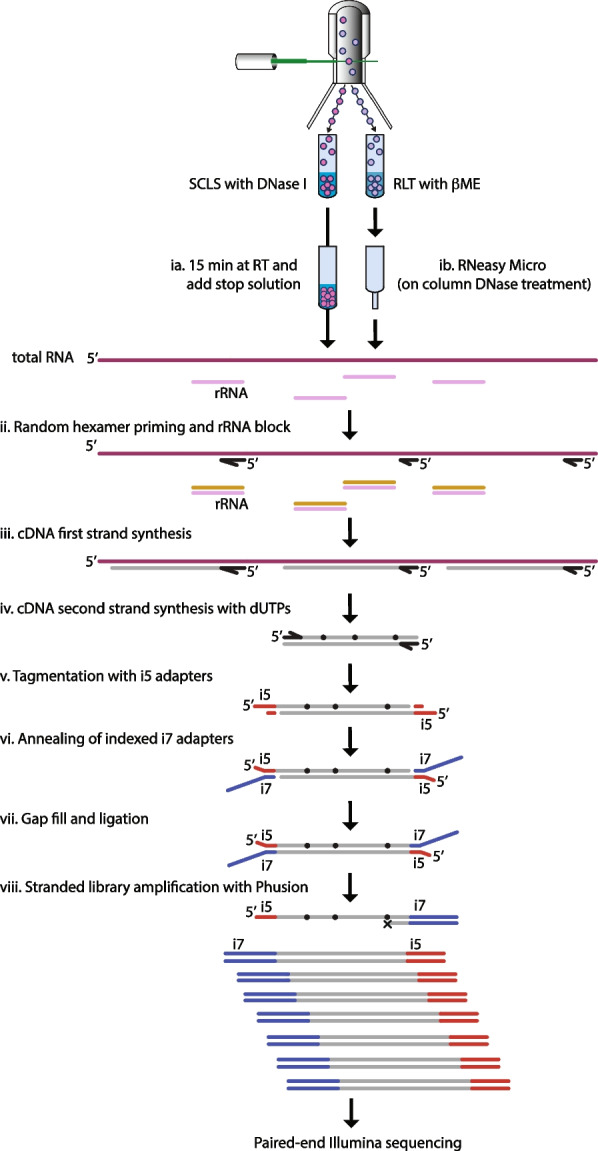
Schematic outline of the T-RHEX-RNAseq protocol. In brief, lysed cells (ia) or total RNA (ib) are subjected to random hexamer priming with rRNA blocking (ii), reverse transcription (iii) and second strand synthesis with dUTP (iv). Subsequently, tagmentation (v) and oligo replacement (vi-vii) are used to introduce adapter sequences before the library is amplified (using dUTP incompatible Phusion) to generate stranded libraries (viii). For details on adapter sequences and primers see Figure S1

Through a series of pilot experiments on purified total RNA, we devised a similar strategy for generating stranded RNAseq libraries using commercially available reagents (data not shown). Together, this formed the core steps of the protocol outlined in Fig. 1. In brief, the NEBNext Ultra II RNA First Strand Synthesis module (NEB) in combination with the NEBNext Ultra II Directional RNA Second Strand Synthesis module (NEB) was used to generate ds cDNA (Fig. 1iii-iv). These reagents were chosen because of the usage of random hexamer priming, stated low input need (1-100 ng prepared RNA) and incorporation of dUTPs in the second strand synthesis. Following second strand synthesis, Tn5 with only i5 compatible adapters was used to tagment the ds cDNA (Fig. 1v and S1). Subsequently, oligo replacement was used to introduce the i7 adapter (Fig. 1vi-vii and S1). For this process to work, the annealed (index containing) i7 replacement oligo needs to be covalently attached to the free 3’- end left by the tagmentation. To achieve this, we used Sulfolobus DNA Polymerase IV (NEB) – which lacks both strand displacement and 5’-3’ exonuclease activity that otherwise could displace or degrade the annealed i7 oligo – in combination with E. coli DNA Ligase (NEB). Together, these enzymes fill the gap left by the tagmentation and seal the remaining nick between the 3’- end of the tagmented ds cDNA and the i7 oligo (Fig. 1vii and S1). Finally, to generate sequencing compatible libraries, we used the Phusion polymerase together with custom i5 completion primers and i7 amplification primers (Fig. 1viii and S1).

### Stranded RNAseq libraries can be generated from very low numbers of FACS sorted cells when RNA purification and dUTP-based degradation steps are omitted

To refine the protocol from the pilot experiments and make it feasible to use low numbers of FACS sorted cells, we next tested if we could omit the RNA purification step and instead directly prepare libraries on lysed cells. At the same time, we wanted to validate that we could simplify the protocol and generate stranded libraries without enzymatically degrading the dUTP containing strand. The rationale behind this being that the High-Fidelity Phusion polymerase (NEB) should generate stranded libraries during the amplification process simply through the inability of the enzyme to read dUTP in the template [27].

**Fig. 2:**
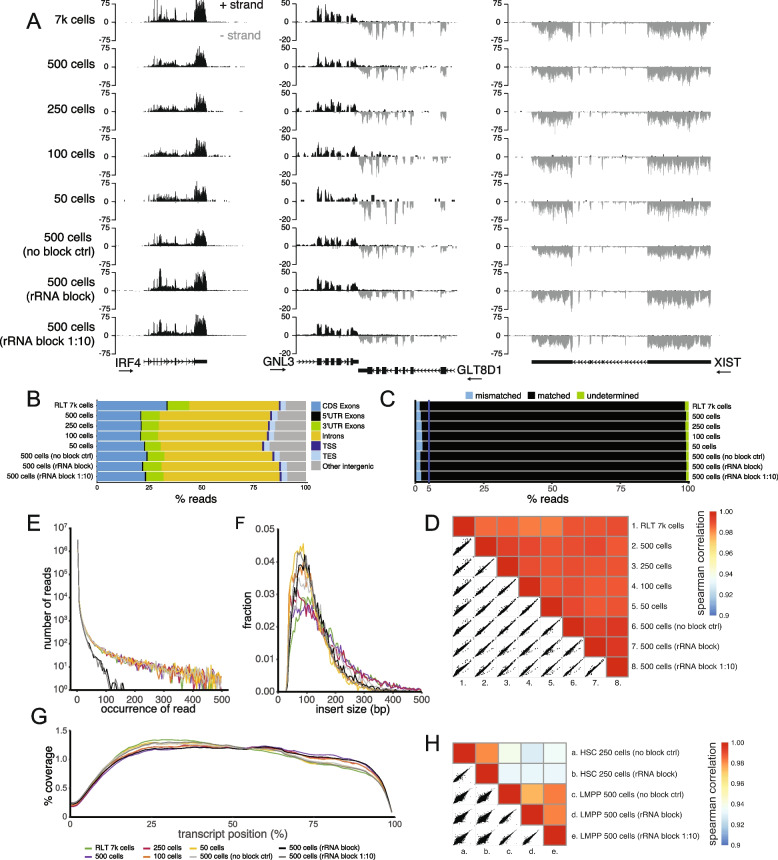
Pilot experiments and establishment of the T-RHEX-RNAseq protocol. **A** Tracks showing reads on the plus and minus strand in the *Irf4*, *Gnl3*, *Glt8d1* and *Xist* genomic regions. Arrows below the gene names indicate the 5’-3’ direction of the transcript. RNAseq libraries were prepared using purified total RNA (7 k MM1.S cells) or directly from the indicated numbers of MM1.S cells lysed in Single cell lysis solution (SCLS). The use of rRNA blocking reagents and dilution of the blocking reagent is indicated in parenthesis. **B** Distribution of reads across genome features. **C** Percentage of reads in exons: localized in a matched or mismatched orientation to transcript; or alternatively being localized in regions with overlapping antiparallel transcripts (undetermined). **D** Spearman correlation and scatter plots of the rlog of gene expression in MM1.S samples. **E** Reoccurrence (duplication rates) of reads in the indicated libraries. Colors used to indicate the individual samples in panels **E**–**G** are shown below panel **G**. **F** Library insert size distributions. **G** Mean distribution of coverage (%) across the length of all transcripts. **H** Spearman correlation and scatter plots of rlog of gene expression in primary mouse hematopoietic stem cells (HSCs) and lymphoid primed multipotent progenitors (LMPPs)

To this end, we FACS sorted a series of progressively lower numbers of cells (500, 250, 100 and 50 MM1.S cells) directly into Single cell lysis solution (SCLS, Invitrogen) with DNase I – to facilitate cell lysis and degradation of genomic DNA – and prepared libraries as outlined in Fig. 1. Inspection of the mapped reads showed that expression showed clear localization of reads to gene bodies (Fig. 2A). As expected from the use of random priming, intronic regions of incompletely spliced transcripts (as seen for *Irf4*) could readily be observed in the library indicating that ongoing transcription and RNA from the nucleus was well captured (Fig. 2A-B).

If the libraries were indeed stranded based on the failure of the polymerase to effectively amplify the dUTP containing strand, the orientation of the reads in relation to transcripts should be maintained. In line with this, tracks clearly showed that the direction of the reads very closely matched the forward or reverse genomic orientation of transcribed genes (Fig. 2A). Looking specifically at *Irf4* and *Xist* (that lack closely located antiparallel promoters that could generate antisense transcripts) these respectively displayed a 182- and 215-fold enrichment of reads matching the orientation of the transcript. On the genome-wide level, we found that between 1.64–2.69% of reads were mismatched in relation to the orientation of the overlapping transcript (Fig. 2C). Hence, adequate strand-specificity can be achieved through the impaired amplification of the dUTP containing strand. To compare this to libraries generated after the degradation of the dUTP containing strand, we re-analyzed the Directional Tn-RNAseq data from Gertz et al., that included a dedicated step to degrade the dUTP containing strand before amplification of the library with Phusion [16]. We found that the Directional Tn-RNAseq libraries displayed between 1.08–1.35% of mismatched reads in exons (Fig. S2). This suggested that degradation of the dUTP containing strand only provided a limited improvement in strand-specificity compared to what is achieved simply through the inability of the Phusion enzyme to amplify dUTP-containing templates.

If present, DNA contamination in RNAseq libraries can be observed as a continuous level of reads across most genomic regions. The clear localization of reads to gene bodies as well as the clearly strand-specific signals in our data both show that no overt DNA contamination is found in the libraries. Hence that the DNase treatment, done as part of the cell lysis, was adequate to hinder genomic DNA from contributing to the library.

In addition, the genome browser tracks (Fig. 2A) indicated that transcriptional profiles were maintained even in the samples generated from the lowest cell numbers. Confirming this notion, library quality was maintained across the full range of input cells with all samples globally having highly similar transcriptome profiles (correlation > 0.98) (Fig. 2D). The libraries also maintained comparable duplication rates (Fig. 2E), library insert sizes (Fig. 2F) mappability (Table S1) and number of expressed genes (Table S1) across the range of progressively lower input cell-numbers.

Random hexamer priming should provide a relatively even coverage across the gene body. Analyzing this, we found that overall the coverage was substantially even with the coefficient of variation for the top 1000 expressed genes being < 0,9 for all MM1.S samples (Fig. 2G and Table S1). A reduction in coverage could however be observed in the first 15% of the 5’-end and in the outermost 3’-end (Fig. 2G). This loss of coverage in the 5’-end can be attributed to the need for Tn5 to integrate in the very outermost part of the 5’-end for it to be represented in the library. Presumably, this effect is mitigated in the 3’-end by the Tn5 integrating in the polyA-tail outside of the transcript specific sequences.

Overall, we concluded that highly strand-specific RNAseq libraries could be generated directly from the lysate of very low numbers of FACS sorted cells without degrading the dUTP containing strand.

### rRNA can effectively be eliminated from the library by blocking prior to reverse transcription

While our initial libraries gave a good representation of expressed RNA, the rRNA contribution to the libraries was substantial in our test experiments (on average 63% of reads) (Table S1). With the rationale that reducing the rRNA contribution to the library before reverse transcription would be preferable to lower the need for deeper sequencing and improve representation of non-rRNA molecules in the library, we tested the QIAseq FastSelect -rRNA HMR kit. While proprietary, the reagent presumably constitutes oligos that block the rRNA molecules from undergoing reverse transcription [10]. To incorporate this in our protocol, we added the QIAseq FastSelect -rRNA HMR reagent at the random hexamer annealing step (Fig. 1ii) and replaced the conventional annealing with stepwise decreases in temperature from 75 °C to 25 °C as per the QIAseq FastSelect protocol. We found that this reduced the rRNA contribution from an average of 63% (range 60–86%) to < 1% of reads (Table S1). Compared to unblocked samples, libraries otherwise maintained overall profiles of mapped reads (Fig. 2A) as well as similarity on the whole-transcriptome level (correlation > 0.98) (Fig. 2D). Generally other library metrics also remained comparable (Fig. 2B, F-G and Table S1). However, the rate of duplication markedly decreased in the blocked samples (Fig. 2E), indicating that the elimination of the rRNA before the reverse transcription improved the representation of original non-rRNA molecules in the library.

With the inclusion of the rRNA blocking at the reverse transcription stage, this formed the basis for our stable version of the T-RHEX-RNAseq protocol (see Methods and Supplemental working protocol).

### T-RHEX-RNAseq can be performed on rare primary cells with high reproducibility

As the T-RHEX-RNAseq protocol was established using the MM1.S cell-line, we next wanted to validate that the protocol could be used to generate libraries on small, mainly resting primary cells that can be expected to contain significantly less RNA per cell. We did this by generating libraries from 250 or 500 FACS sorted primary hematopoietic stem cells (HSCs) and lymphoid primed multipotent progenitors (LMPPs) from mouse bone marrow. Visual inspection of mapped reads indicated that high quality stranded libraries were generated (Fig. S3A) and high correlation was found between replicates (correlation > 0.97) (Fig. 2H). These experiments further supported the conclusion that the overall transcriptional profiles remained unaffected by the inclusion of the rRNA blocking reagent (Fig. 2H) and that blocking the rRNA resulted in improved representation of other RNA species to the library as indicated by the lower duplication rates (Fig. S3B). Further analysis showed that the primary cell libraries displayed similar library metrics to those of the MM1.S libraries with high mapping efficiency (> 74%) and low rRNA contribution (average ≤ 2.2%) in blocked samples (Table S1).

The experiments performed to get proof of principle and establish the T-RHEX-RNAseq method showed that the method could generate data from rare cells. However, the number of samples analyzed was relatively small. To address the reproducibility, we reanalyzed the T-RHEX-RNAseq proof of principle data from the primary cells together with our subsequently generated T-RHEX-RNAseq data from antigen specific CD4 T cells (1000 cells) [28] and hematopoietic stem/progenitor cells (HSPCs) (250–500 cells) [29]. This showed that reproducibility was very high both within the published data sets (correlation > 0.98 and > 0.95 for the T cells and HSPCs respectively) as well as between the HSPC samples from the proof-of-principle experiments and corresponding cells in the subsequently generated hematopoietic stem/progenitor data (correlation > 0.97) (Fig. S4).

Overall, we conclude that directional T-RHEX-RNAseq allows for reproducible generation of high-quality RNA expression data from very low numbers of FACS sorted primary cells.

## Discussion

We have here described stranded T-RHEX-RNAseq, an effective, streamlined and highly reproducible workflow for preparing stranded RNAseq libraries. The simplicity of the protocol, at the core, rests on the use of transposase (loaded with only i5 adapters) in combination with dUTP incorporation to generate the library and maintain strand specificity [16]. Combined with the removal of the need for RNA purification steps as well as dedicated steps to remove rRNA and degrade the dUTP containing strand to achieve strand-specificity this makes a straightforward and easily implemented protocol. Also compared to what could be achieved by scaling up current single cell RNAseq protocols that similarly allow for strand-specific analysis of transcript both with and without poly-adenylation [26], the T-RHEX-RNAseq protocol is significantly less laborious while still achieving high-quality results down to less than one hundred cells.

With a process analogous to that described by Gertz et al., we could effectively prepare libraries from ng amounts of total RNA. However, the direct use of this protocol had limitations for broader implementation including the substantial rRNA contribution to the library caused by the random hexamer priming. Losses of material associated with RNA purification and bead cleanup steps also set direct limits on the lowest possible input cell numbers. Aiming to establish the T-RHEX-RNAseq protocol, we effectively mitigated this limitation by directly preparing the libraries on lysed cells. This allowed us to generate libraries with maintained quality from as little as 50 cells. Considering that quality is maintained with these low input cell numbers, it is feasible that even fewer or single cells could be analyzed using this protocol. The latter in particular, if barcodes could be introduced at the tagmentation step [30] and thereby allow for pooling samples prior to the first bead-cleanup step. While we did not specifically investigate the impact of removing the dUTP degradation and the associated bead cleanup step (prior to library amplification), this likely improved the ability of the T-RHEX-RNAseq protocol to generate libraries on low cell-numbers. This while strand-specificity was still maintained in the library simply through the impaired ability of the High-Fidelity Phusion polymerase to amplify dUTP containing templates [27].

While the use of random hexamer priming on total RNA allows for analyzing a wide range of transcripts, it comes with the disadvantage that rRNA is effectively reverse transcribed. The significant contribution of rRNA to the library seen in our initial tests hence was a significant hindrance from sequencing RNA of interest in a cost-effective manner. Given the scarce input material and the complexity of depleting the rRNA derived part of the final library, we reasoned that blocking rRNA from undergoing reverse transcription would by far be the simplest and most effective solution to implement. In the T-RHEX-RNAseq protocol, we found that this approach substantially mitigated the issue of rRNA contributing to the library. This not only lowered the sequencing needed to acquire adequate depth for analyzing RNA expression but interestingly also improved the representation of RNA of interest in the library. The latter suggests that this approach of blocking rRNA before reverse transcription could be generally beneficial for RNAseq approaches using cell lysates or total RNA as starting material.

In terms of implementation, the T-RHEX-RNAseq can be performed using commercially available reagents and offers flexible use of purified RNA or cells collected in SCLS. Practically, storing cells in SCLS in individual PCR tubes – that can be attached to a holder to generate a 96-well plate – offers an adaptable solution where samples easily can be selected for analysis and collected into larger batches that can then be processed in a semi high-throughput manner in 96-well format. While we have commonly generated T-RHEX-RNAseq libraries over two days, it is practically possible for an experienced user to generate libraries in a single day and then perform sequencing on the NextSeq 500/550/2000 systems the following day. This makes it possible to start processing the sequencing data less than 48 h after the start of the library preparation which overall makes this a time efficient process. In terms of performing cost efficient sequencing, the smallest versions of NextSeq sequencing kits (75 or 50 cycles depending on system) are both sufficient for paired-end 41 sequencing, which gives good coverage for most library inserts. While longer reads likely would improve mapping efficiency, this approach provides a good trade-off between data quality, cost and time spent with the sequencing taking approximately 12 h.

Hence, overall T-RHEX-RNAseq offers an easily implemented and convenient solution for generating stranded RNAseq libraries from nanograms of purified total RNA down to very low numbers of FACS sorted cells.

## Conclusion

We here describe T-RHEX-RNAseq, an easy-to-use protocol for generating stranded RNAseq libraries from low numbers of FACS sorted cells. The protocol uses the simplicity offered by tagmentation and takes advantage of random hexamer priming while avoiding limitations caused by rRNA removal and RNA purification steps. This offers a time efficient and reproducible method for analyzing complete transcriptomes, that is compatible with applications ranging from analysis of rare cells to large scale projects requiring automation.

## Methods

### Cells and FACS sorting

MM1.S (ATCC #CRL-2974) was cultured in RPMI-1640 with GlutaMAX and HEPES supplemented with 10% fetal calf serum and 1% penicillin–streptomycin. Defined numbers of MM1.S cells were FACS sorted on a FACSARIA IIu (BD Biosciences) using propidium iodine to exclude dead cells. Primary mouse HSCs (LIN- SCA1 + KIT + CD48- CD150high) and LMPPs (LIN- SCA1 + KIT + FLT3high CD150-) were FACS sorted from the bone marrow of a 12-week-old female C57BL/6 J mouse (directly sacrificed using cervical dislocation in concordance with AVMA and local regulations) as previously described [24]. Animals were bred and maintained at the Preclinical Laboratory, Karolinska University Hospital and experiments were approved by the regional ethical committee. To perform RNAseq on lysed cells, cells were FACS sorted directly into 5 or 10 μl Single cell lysis solution (SCLS; Single cell lysis kit Invitrogen, cat# 4458235) with DNase I according to manufacturer’s instructions with the exception that the lysis reaction was incubated for 15 min at room temperature (RT) before adding stop solution. To perform RNAseq on purified total RNA, cells were FACS sorted into RLT buffer with β-mercaptoethanol. RNA was extracted using the RNeasy Micro Kit (Qiagen, cat#74004) with on-column DNase I treatment according to manufacturer’s instructions. Both samples in SCLS and RLT were snap frozen on dry ice and stored at -80 °C until use.

### Tn5 with i5 adapters

Tn5 loaded with only i5 compatible adapters was kindly provided by Prof. Rickard Sandberg or generated by loading purified un-loaded Tn5 tagmentase (Diagenode cat#C01070010) with adapters according to the manufacturer’s instructions (see Fig. S1 and working protocol for adapter sequences).

### T-RHEX-RNAseq

Double stranded cDNA was prepared from lysed cells in SCLS or purified total RNA using NEBNext Ultra II RNA First Strand Synthesis (NEB cat#E7771) and NEBNext Ultra II Directional RNA Second Strand Synthesis (NEB cat#E7550) modules according to manufacturer’s instructions. In rRNA blocked samples, QIAseq FastSelect -rRNA HMR kit reagent (Qiagen cat# 334386) was added to the first strand cDNA synthesis reaction according to manufacturer’s instructions with the exception that only one tenth the amount was used for the majority of libraries. Stranded library preparation was subsequently initiated by adding tagmentation buffer (50 mM tris acetate, 25 mM Mg acetate, 50% dimethylformamide) and Tn5 with i5 adapters to ds cDNA. After 5 min at 55 °C, the reaction was stopped by incubating with SDS for 5 min at room temperature and samples were purified using AmPure XP beads at a ratio sample:beads of 1:1.2 and eluted in water. Introduction of indexes through oligo replacement [16] was carried out by adding custom made i7 replacement oligos and gap fill buffer (165 mM tris acetate, 330 mM potassium acetate, 50 mM Mg acetate, 2.5 mM DTT, 1 mM beta-NAD and 1.25 mM each of dATP, dCTP, dGTP and dTTP). After 30 min incubation at 37 °C, gap fill enzymes were added (1U Sulfolobus DNA Polymerase IV NEB cat# M0327S and 10U E. coli DNA Ligase NEB cat# M0205L) and incubation continued for an additional 30 min at 37 °C. Samples were purified using AmPure XP beads at a ratio sample:beads of 1:1.2 and eluted in water. Libraries were subsequently PCR amplified using Phusion HF PCR Master Mix (NEB) with the following PCR program: 95 °C 2 min; followed by 16 cycles of 94 °C 10 s, 60 °C 30 s and 72 °C 1 min. Library cleanup was done using AmPure XP beads at a ratio sample:beads of 1:0.9. DNA concentrations in purified samples were measured using the Qubit dsDNA HS Kit (Invitrogen). Barcoded libraries were pooled and paired-end sequenced (2 × 41 cycles) using the Illumina platform (NextSeq500, Illumina).

### Analysis of RNAseq data

Samples were analyzed using the nf-core/rnaseq pipeline (v.3.3) [31, 32] with the –remove_ribo_rna option and the default rRNA database. Mouse and human samples were aligned to the mm10 and hg38 reference genome, respectively. Mapping rates, GC content and rRNA contribution to the libraries were derived from nf-core via MultiQC [33]. The number of expressed genes was calculated based on TPM values obtained from nf-core. Median coefficient of variation (CV) for the coverage across the 1000 highest expressed genes was calculated using Picard Tools’ (v2.27.5) CollectRnaSeqMetrics. For visualization of read coverage, tracks obtained from nf-core were normalized to 10^7^ mapped reads and uploaded to the UCSC genome browser [34]. Read distribution over genome features was derived from the nf-core MultiQC report. Inferred strandedness was determined using the RSeqQC package (v.2.6.4) considering reads overlapping known exons. To perform correlation analysis between samples, per gene read counts were derived from nf-core, read counts of expressed genes (≥ 30 reads in ≥ 3 human or mouse samples) normalized using rlog transformation, and the spearman correlation was calculated using R (v4.1.1). To compare duplication rates between samples, bam files were downsampled to the same number of mapped reads and duplication rates determined using the read_duplication.py function from the RSeqQC package. R was then used to calculate the occurrence of duplicated reads for each 5-step increment. Library insert sizes were derived from nf-core and binned into 5 bp bins. Coverage across the length of the gene body was derived from the nf-core MultiQC report and re-calculated to % of total coverage. Data was visualized using R and the ggplot2 package (v3.3.6).

## Supplementary Information

**Additional file 1:** **Figure S1.** Schematic overview of the T-RHEX-RNAseq protocol outlining adapters, adapter introduction and primers used to amplify the library. In brief, double stranded cDNA reverse transcription with dUTP incorporated during the second strand synthesis is subjected to tagmentation with Tn5 loaded with i5 adapters. The i7 adapters are introduced by annealing an i7 oligo to the covalently attached part of the i5 adapter. Subsequently, gap fill in combination with ligation is used to covalently attach the i7 adapter. As Phusion is unable to utilize the dUTP containing strand as a template, stranded libraries are then generated by amplification using Pr2 in combination with the i5 completion primer. **Figure S2.** Strand-specificity of Tn-RNAseq and Directional Tn-RNAseq libraries. (A). Percentage of reads in exons: localized in a matched or mismatched orientation to transcript; or alternatively being localized in regions with overlapping antiparallel transcripts (undetermined). The data from Gertz et al., [1] was downloaded and processed using nf-core and strand-specificity evaluated using RSeq QC. **Figure S3.** Tracks and duplication rates of T-RHEX-RNAseq libraries from primary hematopoietic stem- and progenitor cells. (A) Tracks showing plus and minus strand reads in the *Neat1, Kit, Hspd1 and Hspe1* genomic regions in primary mouse hematopoietic stem cells (HSCs) and lymphoid primed multipotent progenitors (LMPPs). Arrows below the gene names indicate the 5’-3’ direction of the transcript. RNAseq libraries were prepared directly from the indicated numbers of cells lysed in Single cell lysis solution (SCLS). The use of rRNA blocking reagents and dilution of the blocking reagent is indicated in parenthesis. (B) Reoccurrence (duplication rates) of reads in the indicated libraries. **Figure S4.** T-RHEX-RNAseq provides highly reproducible data. Spearman correlation between rlog of gene expression in samples generated from the indicated population. The use of rRNA blocking reagents and dilution of the blocking reagent is indicated in parenthesis. Hematopoietic stem cell (HSC with or without CD49b expression); Multipotent progenitor (MPP with no or low CD150 expression), lymphoid primed multipotent progenitors (LMPPs); granulocyte/monocyte progenitors (GMP); and antigen specific CD4 T cells (T, from wild-type or Bhlhe40 knockout mice). Data is from proof-of-principle experiments (HSC and LMPP; 250 and 500 cells respectively) or the subsequently generated T-RHEX-RNAseq data from antigen specific CD4 T cells (1000 cells) [1] and hematopoietic stem/progenitor cells (HSPCs; 250-500 cells) [2]. **Table S1.** Sample metrics and QC. **Supplemental** **working** **protocol**.

## Data Availability

RNAseq data is available from: the European Nucleotide Archive (ENA) under accession numbers PRJEB56375 (T-RHEX-RNAseq proof-of-principle experiments on MM1.S and HSPCs) and PRJEB47791 (HSPCs) [24]; and the Gene Expression Omnibus (GEO) under accession number GSE173673 (antigen specific CD4 T cells) [23].
