# Supplementary material for: T-RHEX-RNAseq – a tagmentation-based, rRNA blocked, random hexamer primed RNAseq method for generating stranded RNAseq libraries directly from very low numbers of lysed cells
Source: BMC Genomics. 2023 Apr 17;24:205. doi: 10.1186/s12864-023-09279-4 (PMC10111750; doi:10.1186/s12864-023-09279-4)
Supplement: Supplementary file 1 — Additional file 1: Figure S1. Schematic overview of the T-RHEX-RNAseq protocol outlining adapters, adapter introduction and primers used to amplify the library. In brief, double stranded cDNA reverse transcription with dUTP incorporated during the second strand synthesis is subjected to tagmentation with Tn5 loaded with i5 adapters. The i7 adapters are introduced by annealing an i7 oligo to the covalently attached part of the i5 adapter. Subsequently, gap fill in combination with ligation is used to covalently attach the i7 adapter. As Phusion is unable to utilize the dUTP containing strand as a template, stranded libraries are then generated by amplification using Pr2 in combination with the i5 completion primer. Figure S2. Strand-specificity of Tn-RNAseq and Directional Tn-RNAseq libraries. (A). Percentage of reads in exons: localized in a matched or mismatched orientation to transcript; or alternatively being localized in regions with overlapping antiparallel transcripts (undetermined). The data from Gertz et al., [1] was downloaded and processed using nf-core and strand-specificity evaluated using RSeq QC. Figure S3. Tracks and duplication rates of T-RHEX-RNAseq libraries from primary hematopoietic stem- and progenitor cells. (A) Tracks showing plus and minus strand reads in the Neat1, Kit, Hspd1 and Hspe1 genomic regions in primary mouse hematopoietic stem cells (HSCs) and lymphoid primed multipotent progenitors (LMPPs). Arrows below the gene names indicate the 5’-3’ direction of the transcript. RNAseq libraries were prepared directly from the indicated numbers of cells lysed in Single cell lysis solution (SCLS). The use of rRNA blocking reagents and dilution of the blocking reagent is indicated in parenthesis. (B) Reoccurrence (duplication rates) of reads in the indicated libraries. Figure S4. T-RHEX-RNAseq provides highly reproducible data. Spearman correlation between rlog of gene expression in samples generated from the indicated population. The use of rRNA [file 12864_2023_9279_MOESM1_ESM.pdf]

SUPPLEMENTAL INFORMATION

Figure S1

Figure S2

Figure S3

Figure S4

Table S1

Working protocol

Fig. S1

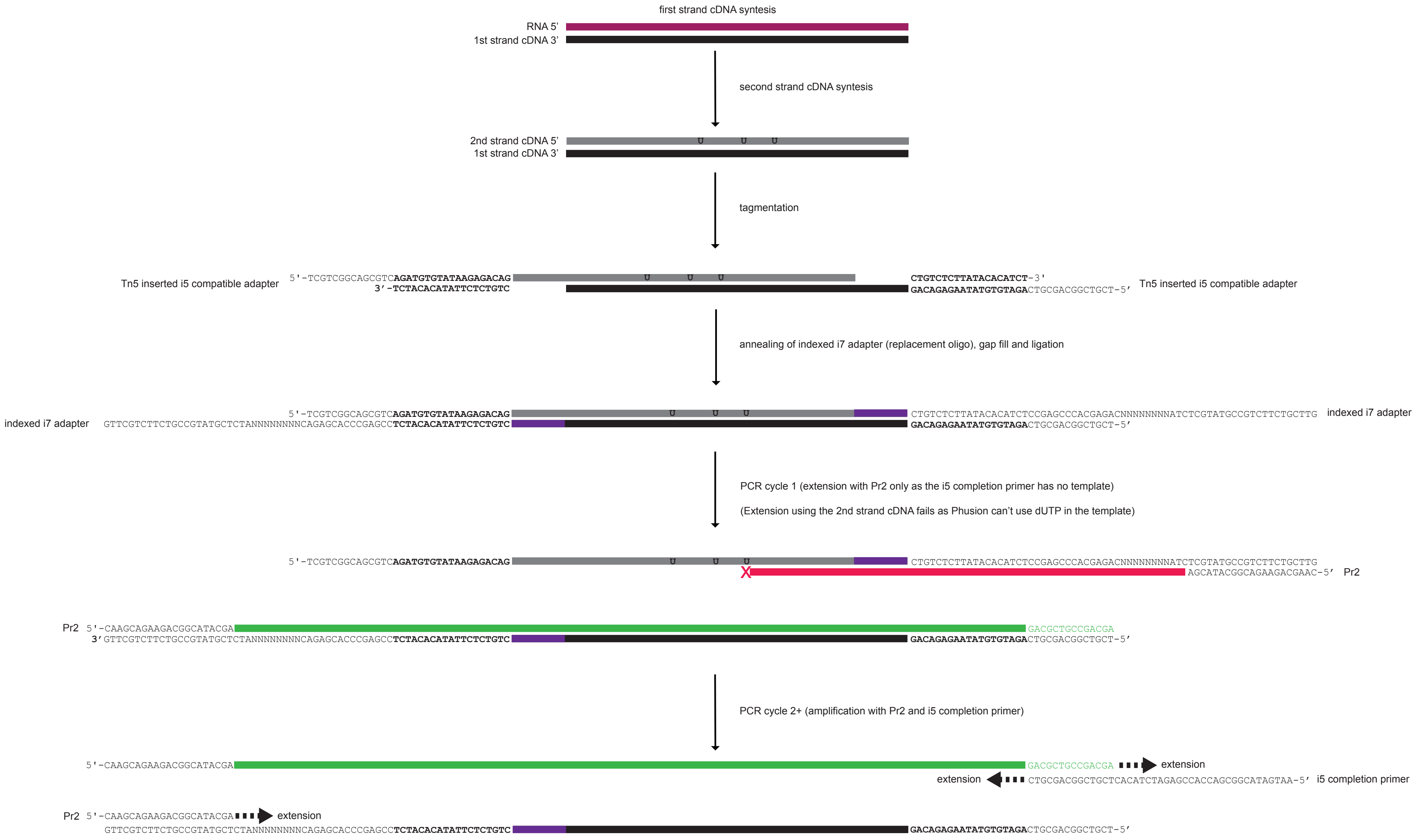

first strand cDNA syntesis
RNA 5'
1st strand cDNA 3'
second strand cDNA syntesis
2nd strand cDNA 5'
1st strand cDNA 3'
tagmentation
Tn5 inserted i5 compatible adapter
5'-TCGTCGGCAGCGTCAGATGTGTATAAGAGACAG
3'-TCTACACATATTCTCTGTC
CTGTCTCTTATACACATCT-3'
GACAGAGAATATGTGTAGACTGCGACGGCTGCT-5'
Tn5 inserted i5 compatible adapter
annealing of indexed i7 adapter (replacement oligo), gap fill and ligation
indexed i7 adapter
5'-TCGTCGGCAGCGTCAGATGTGTATAAGAGACAG
GTTCGTCTTCTGCCGTATGCTCTANNNNNNNNNCAGAGCACCCGAGCCTCTACACATATTCTCTGTC
CTGTCTCTTATACACATCTCCGAGCCCACGAGACNNNNNNNNNATCTCGTATGCCGTCTTCTGCTTG
GACAGAGAATATGTGTAGACTGCGACGGCTGCT-5'
indexed i7 adapter
PCR cycle 1 (extension with Pr2 only as the i5 completion primer has no template)
(Extension using the 2nd strand cDNA fails as Phusion can't use dUTP in the template)
5'-TCGTCGGCAGCGTCAGATGTGTATAAGAGACAG
CTGTCTCTTATACACATCTCCGAGCCCACGAGACNNNNNNNNNATCTCGTATGCCGTCTTCTGCTTG
AGCATACGGCAGAAGACGAAC-5'
Pr2
Pr2
5'-CAAGCAGAAGACGGCATACGA
GACGCTGCCGACGA
3'GTTCGTCTTCTGCCGTATGCTCTANNNNNNNNNCAGAGCACCCGAGCCTCTACACATATTCTCTGTC
GACAGAGAATATGTGTAGACTGCGACGGCTGCT-5'
PCR cycle 2+ (amplification with Pr2 and i5 completion primer)
5'-CAAGCAGAAGACGGCATACGA
GACGCTGCCGACGA
extension
extension
CTGCGACGGCTGCTCACATCTAGAGCCACCAGCGGCATAGTAA-5'
i5 completion primer
Pr2
5'-CAAGCAGAAGACGGCATACGA
extension
GTTCGTCTTCTGCCGTATGCTCTANNNNNNNNNCAGAGCACCCGAGCCTCTACACATATTCTCTGTC
GACAGAGAATATGTGTAGACTGCGACGGCTGCT-5'

Figure S1. Schematic overview of the T-RHEX-RNAseq protocol outlining adapters, adapter introduction and primers used to amplify the library. In brief, double stranded cDNA reverse transcription with dUTP incorporated during the second strand synthesis is subjected to tagmentation with Tn5 loaded with i5 adapters. The i7 adapters are introduced by annealing an i7 oligo to the covalently attached part of the i5 adapter. Subsequently, gap fill in combination with ligation is used to covalently attach the i7 adapter. As Phusion is unable to utilize the dUTP containing strand as a template, stranded libraries are then generated by amplification using Pr2 in combination with the i5 completion primer.

Fig. S2

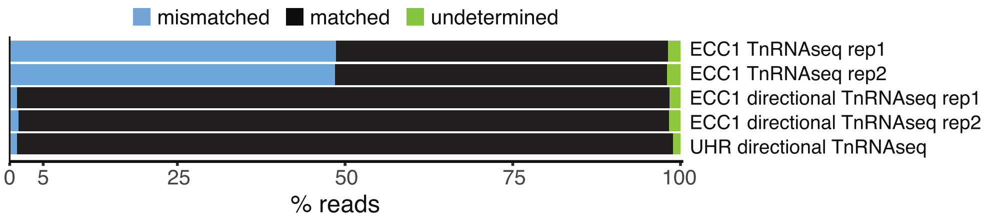

mismatched
matched
undetermined
ECC1 TnRNAseq rep1
ECC1 TnRNAseq rep2
ECC1 directional TnRNAseq rep1
ECC1 directional TnRNAseq rep2
UHR directional TnRNAseq
0
5
25
50
75
100
% reads

Figure S2. Strand-specificity of Tn-RNAseq and Directional Tn-RNAseq libraries. (A) Percentage of reads in exons: localized in a matched or mismatched orientation to transcript; or alternatively being localized in regions with overlapping antiparallel transcripts (undetermined). The data from Gertz et al., [1] was downloaded and processed using nf-core and strand-specificity evaluated using RSeqQC.

[1] Gertz J, Varley KE, Davis NS, Baas BJ, Goryshin IY, Vaidyanathan R, et al. Transposase mediated construction of RNA-seq libraries. Genome Res. 2012;22:134–41.

Fig. S3

A

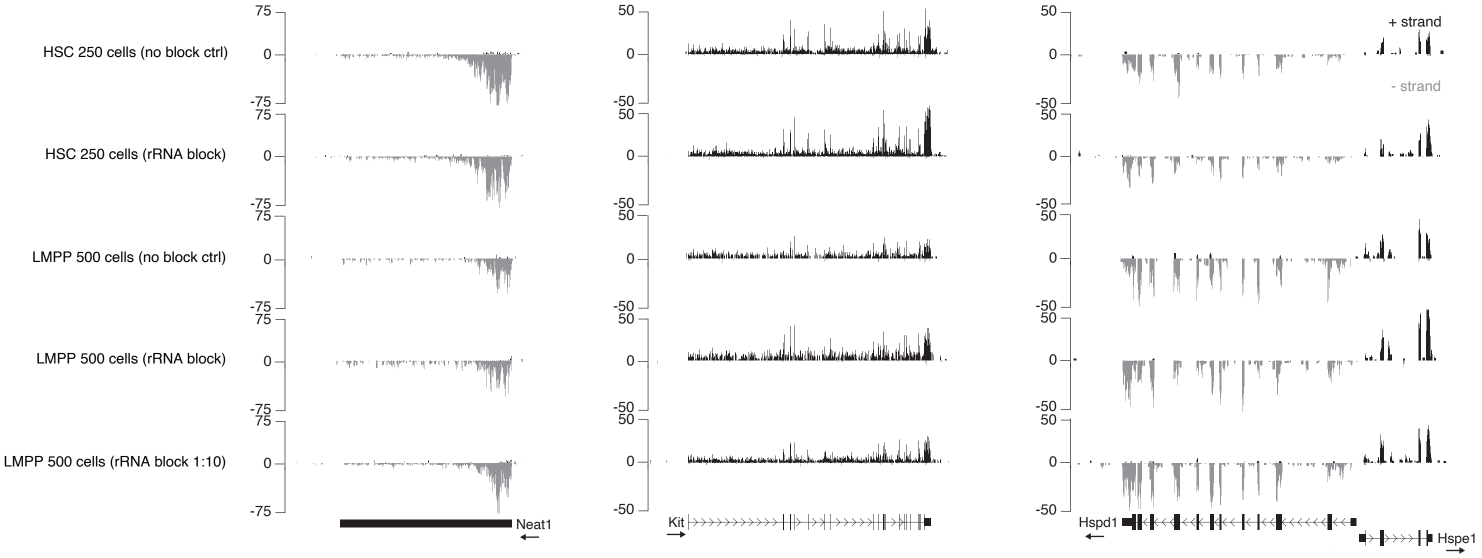
HSC 250 cells (no block ctrl)
HSC 250 cells (rRNA block)
LMPP 500 cells (no block ctrl)
LMPP 500 cells (rRNA block)
LMPP 500 cells (rRNA block 1:10)
+ strand
- strand
Neat1
Kit
Hspd1
Hspe1

B

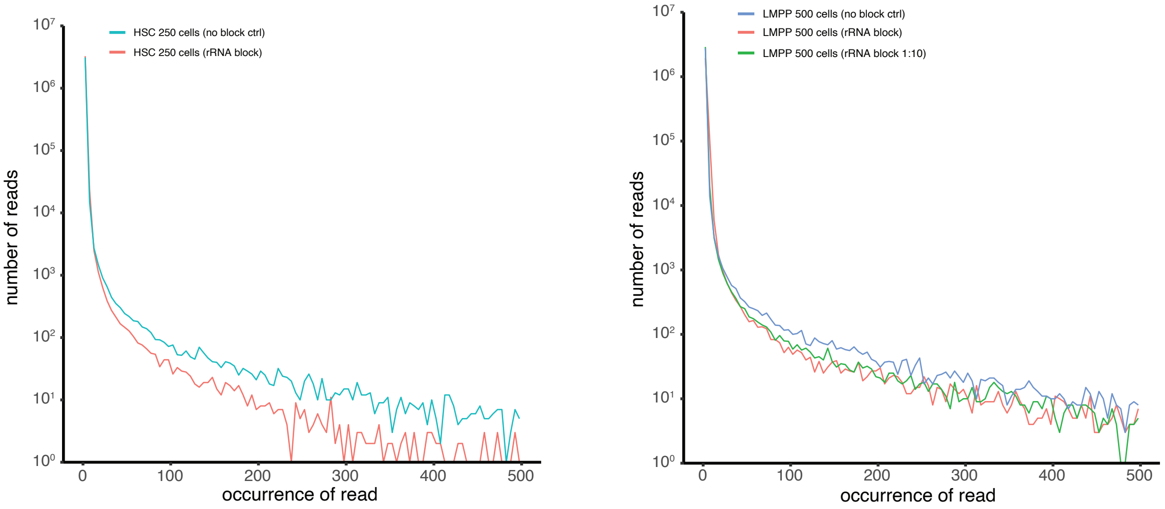
HSC 250 cells (no block ctrl)
HSC 250 cells (rRNA block)
number of reads
occurrence of read
LMPP 500 cells (no block ctrl)
LMPP 500 cells (rRNA block)
LMPP 500 cells (rRNA block 1:10)
number of reads
occurrence of read

Figure S3. Tracks and duplication rates of T-RHEX-RNAseq libraries from primary hematopoietic stem- and progenitor cells. (A) Tracks showing plus and minus strand reads in the *Neat1*, *Kit*, *Hspd1* and *Hspe1* genomic regions in primary mouse hematopoietic stem cells (HSCs) and lymphoid primed multipotent progenitors (LMPPs). Arrows below the gene names indicate the 5'-3' direction of the transcript. RNAseq libraries were prepared directly from the indicated numbers of cells lysed in Single cell lysis solution (SCLS). The use of rRNA blocking reagents and dilution of the blocking reagent is indicated in parenthesis. (B) Reoccurrence (duplication rates) of reads in the indicated libraries.

Fig. S4

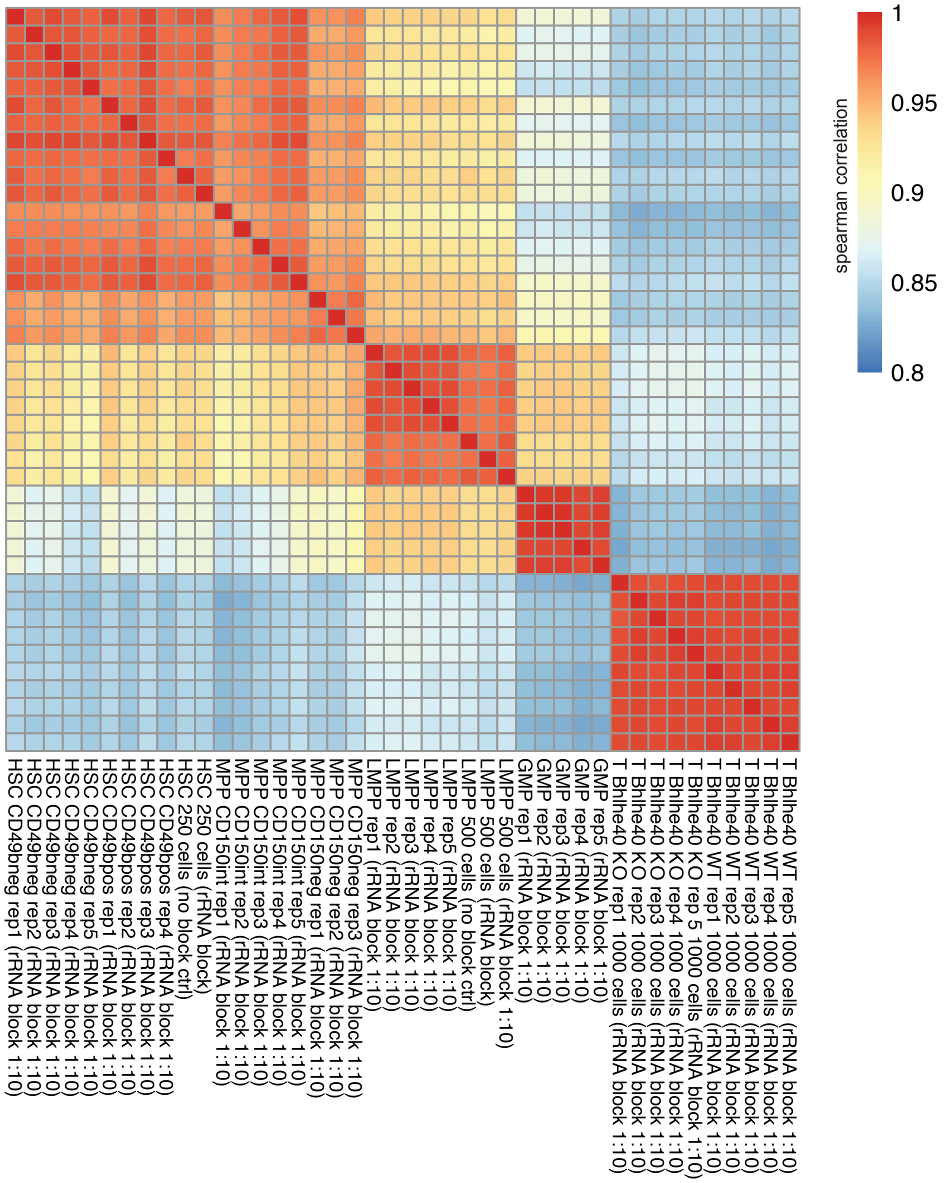
spearman correlation
1
0.95
0.9
0.85
0.8
HSC CD49bneg rep1 (rRNA block 1:10)
HSC CD49bneg rep2 (rRNA block 1:10)
HSC CD49bneg rep3 (rRNA block 1:10)
HSC CD49bneg rep4 (rRNA block 1:10)
HSC CD49bneg rep5 (rRNA block 1:10)
HSC CD49bpos rep1 (rRNA block 1:10)
HSC CD49bpos rep2 (rRNA block 1:10)
HSC CD49bpos rep3 (rRNA block 1:10)
HSC CD49bpos rep4 (rRNA block 1:10)
HSC 250 cells (no block ctrl)
HSC 250 cells (rRNA block)
MPP CD150int rep1 (rRNA block 1:10)
MPP CD150int rep2 (rRNA block 1:10)
MPP CD150int rep3 (rRNA block 1:10)
MPP CD150int rep4 (rRNA block 1:10)
MPP CD150int rep5 (rRNA block 1:10)
MPP CD150neg rep1 (rRNA block 1:10)
MPP CD150neg rep2 (rRNA block 1:10)
MPP CD150neg rep3 (rRNA block 1:10)
LMPP rep1 (rRNA block 1:10)
LMPP rep2 (rRNA block 1:10)
LMPP rep3 (rRNA block 1:10)
LMPP rep4 (rRNA block 1:10)
LMPP rep5 (rRNA block 1:10)
LMPP 500 cells (no block ctrl)
LMPP 500 cells (rRNA block)
LMPP 500 cells (rRNA block 1:10)
GMP rep1 (rRNA block 1:10)
GMP rep2 (rRNA block 1:10)
GMP rep3 (rRNA block 1:10)
GMP rep4 (rRNA block 1:10)
GMP rep5 (rRNA block 1:10)
T Bhlhe40 KO rep1 1000 cells (rRNA block 1:10)
T Bhlhe40 KO rep2 1000 cells (rRNA block 1:10)
T Bhlhe40 KO rep3 1000 cells (rRNA block 1:10)
T Bhlhe40 KO rep4 1000 cells (rRNA block 1:10)
T Bhlhe40 KO rep 5 1000 cells (rRNA block 1:10)
T Bhlhe40 WT rep1 1000 cells (rRNA block 1:10)
T Bhlhe40 WT rep2 1000 cells (rRNA block 1:10)
T Bhlhe40 WT rep3 1000 cells (rRNA block 1:10)
T Bhlhe40 WT rep4 1000 cells (rRNA block 1:10)
T Bhlhe40 WT rep5 1000 cells (rRNA block 1:10)

Figure S4. T-RHEX-RNAseq provides highly reproducible data. Spearman correlation between rlog of gene expression in samples generated from the indicated population. The use of rRNA blocking reagents and dilution of the blocking reagent is indicated in parenthesis. Hematopoietic stem cell (HSC with or without CD49b expression); Multipotent progenitor (MPP with no or low CD150 expression), lymphoid primed multipotent progenitors (LMPPs); granulocyte/monocyte progenitors (GMP); and antigen specific CD4 T cells (T, from wild-type or Bhlhe40 knockout mice). Data is from proof-of-principle experiments (HSC and LMPP; 250 and 500 cells respectively) or the subsequently generated T-RHEX-RNAseq data from antigen specific CD4 T cells (1000 cells) [1] and hematopoietic stem/progenitor cells (HSPCs; 250-500 cells) [2].

[1] Rauschmeier R, Reinhardt A, Gustafsson C, Glaros V, Artemov AV, Dunst J, et al. Bhlhe40 function in activated B and TFH cells restrains the GC reaction and prevents lymphomagenesis. J Exp Med. 2021;219:e20211406.

[2] Somuncular E, Hauenstein J, Khalkar P, Johansson A-S, Dumral Ö, Frengen NS, et al. CD49b identifies functionally and epigenetically distinct subsets of lineage-biased hematopoietic stem cells. Stem Cell Rep. 2022;17:1546–60.

Table S1. Sample metrics and QC.

Sample	million read pairs	million (non rRNA) read pairs aligned	% aligned of total read pairs	% aligned of non rRNA read pairs	rRNA block	% rRNA of total read pairs	GC content (in all reads)	# genes expressed (≥1TPM)	evenness of coverage (median CV)
MM1.S RLT 7k cells	44,6	12,7	28,5%	86%	n/a	66%	50%	12554	0,65
MM1.S SCLS 500 cells	38,7	11,6	30,0%	82%	n/a	62%	49%	12146	0,64
MM1.S SCLS 250 cells	21,0	5,9	28,1%	78%	n/a	60%	49%	11875	0,73
MM1.S SCLS 100 cells	17,4	4,4	25,3%	77%	n/a	64%	50%	11631	0,80
MM1.S SCLS 50 cells	7,9	1,7	21,5%	69%	n/a	64%	49%	11248	0,90
MM1.S SCLS 500 cells (no block ctrl)	34,5	7,5	21,7%	83%	no	72%	52%	11921	0,58
MM1.S SCLS 500 cells (rRNA block)	39,1	34,1	87,2%	88%	yes	0,3%	41%	12492	0,55
MM1.S SCLS 500 cells (rRNA block 1:10)	45,7	38,6	84,5%	85%	yes, diluted 1:10	0,8%	42%	12232	0,52
mouse LMPP SCLS 500 cells (no block ctrl)	39,7	2,7	6,8%	64%	no	86%	54%	11575	0,87
mouse LMPP SCLS 500 cells (rRNA block)	31,2	23,0	73,7%	74%	yes	0,7%	48%	11514	0,92
mouse LMPP SCLS 500 cells (rRNA block 1:10)	26,2	18,7	71,4%	76%	yes, diluted 1:10	5,6%	50%	11758	0,77
mouse HSC SCLS 250 cells (no block ctrl)	38,0	6,2	16,3%	62%	no	70%	51%	12394	0,77
mouse HSC SCLS 250 cells (rRNA block)	33,3	27,4	82,3%	83%	yes	0,3%	44%	12457	0,73

Charlotte Gustafsson 2022-07-07 v3

T-RHEX-RNAseq on 250-1000 cells sorted into Single cell lysis solution

Cell sorting into SCLS (Single cell lysis kit Invitrogen, Thermo Fisher Scientific cat# 4458235)

1. Use tubes that fit your sorter, for example Biorad 0.2ml flat cap strip tubes (cat# TLS0801 and TCS0803[1]) and make sure the stream hits the lysis solution at the bottom of the tube.
2. Sort up to 1000 cells into tubes containing 0.5µl DNase I and 4.5µl Single cell lysis solution. Keep tubes at 4°C during sorting.
3. Incubate at RT 15min (no mixing is required).[2]
4. Add 0.5µl Single cell stop solution. No mixing is required.
5. Incubate at RT 2min.
6. Snap freeze on dry ice.
7. Transfer to -80°C until further use.

Primer annealing and rRNA block (NEBNext Ultra™ II RNA First Strand Synthesis Module, NEB cat#E7771; QIAseq FastSelect -rRNA HMR kit, Qiagen cat# 334386)

Work on ice. In a PCR tube, combine:

5µl Lysed cells
4µl NEBNext 1st strand synthesis reaction buffer (lilac)
1µl NEBNext Random hexamer primers (lilac)
<u>1µl Qiagen FastSelect rRNA HMR reagent[3]</u>
Total 11µl

Flick the tube/strip, quickly spin down. Incubate sample in a PCR machine using the PRIME + rRNA-BLOCK program.

PRIME + rRNA-BLOCK	
Lid 105°C	
75°C	2 min
70°C	2 min
65°C	2 min
60°C	2 min
55°C	2 min
37°C	2 min
25°C	2 min
4°C	hold

First strand cDNA synthesis (NEBNext Ultra™ II RNA First Strand Synthesis Module, NEB cat#E7771)

Work on ice. Continuing in the same PCR tube, combine:

11µl primed and blocked RNA (from previous step)
8µl NEBNext Strand specificity reagent (white/brown tube)
<u>2µl NEBNext First strand synthesis enzyme mix (lilac)</u>
Total 21µl

Flick the tube/strip, quickly spin down. Incubate sample in a PCR machine using the cDNA1STR program.

cDNA1STR	
Lid 105°C	
25°C	10 min
42°C	50 min
70°C	15 min
4°C	hold

Second strand cDNA synthesis (NEBNext Ultra™ II Directional RNA Second Strand Synthesis Module, NEB Cat# E7550)

Work on ice. Continuing in the same PCR tube combine:

21µl cDNA sample (from previous step)
8µl NEBNext 2nd strand synthesis reaction buffer dUTP (orange)
4µl NEBNext 2nd strand synthesis enzyme mix (orange)
<u>48µl nuclease free water</u>
Total 81µl

cDNA2STR	
Lid 40°C	
16°C	60 min
80°C	15 min
4°C	hold

Flick the tube/strip, quickly spin down. Incubate sample in a PCR machine using the cDNA2STR program.
(Safe stopping point. Samples can be kept overnight at 4°C.)

1. Tubes can be individually fastened to 96-well racks (Biorad cat#TRC9601) for easy handling in plate format.
2. We use a prolonged incubation at RT compared to kit protocol, to reduce DNA content seen in some samples sorted with cooling.
3. 1:10 dilution of HMR reagent in water gives similar results.

Tagmentation (using Tn5 enzyme containing only i5 adapters)

Work on ice. Continuing in the same PCR tube combine:

81µl double stranded (ds) cDNA sample (from previous step)
20µl 5X Tn5 Tagmentation buffer (home-made, see reagents section)
<u>1µl Tn5 with i5 adapters (see reagents section)</u>
Total 102µl

dscDNATN5	
Lid 105°C	
55°C	5 min
4°C	hold

Flick the tube/strip, quickly spin down. Incubate sample in a PCR machine using the dscDNATN5 program.

Add 5µl Tagmentation Stop Solution (1% SDS, see reagents section), mix and quickly spin down. Incubate at RT 5min.

Bead cleanup of library (Ampure XP beads, Beckman Coulter cat#A63881; DynaMag™-96 Side Magnet, Thermo Fisher Scientific cat#12331D)

Let beads adjust to RT for 15-30min.

1) Mix Ampure beads thoroughly, add 130µl beads (1.2:1) to sample (107µl). Set pipette to 230µl and pipette x10 in tube. Incubate at RT for 5 min.
2) Place sample on magnet for 5 min until supernatant is clear.
3) Gently remove and discard 235µl of the supernatant without disturbing the beads.
4) With sample still on magnet, add 200µl 80% EtOH, leave for 30s, then gently discard all supernatant. Repeat once for a total of two washings.
5) Quickly spin down tube, put back on magnet and remove all remaining EtOH. (Optionally, if not using a spindown, let beads air dry for 3 min.) Do not let beads over-dry.
6) Resuspend sample in 15µl water, incubate at RT for 2 min.
7) Place sample on magnet for 3-5 min until supernatant is clear.
8) Transfer 14µl of clear supernatant to a new PCR tube (preferably containing index and gap fill reagents, see next step below).

Oligo replacement and gap fill

Thaw oligos and buffer at RT and keep on ice. Gap fill buffer and Replacement index can be combined in a new tube during the above Ampure cleanup. Then add supernatant directly into tube with reagents.

14µl tagmented sample (from previous step)
4µl Gap fill buffer (home-made, see reagents section)
<u>1µl Replacement index i7 oligo (40µM, index Ad2.1-Ad2.24, see reagents section)</u>
Total 19µl

Pipette or flick the tube/strip, quickly spin down. Incubate sample in a PCR machine using the RNAGF program.

RNAGF	
Lid 105°C	
45°C	1 min
37°C	30 min
PAUSE	
37°C	30 min
4°C	hold

After 30 min pause program and add:
<u>1.5µl Gap fill and ligation enzyme mix (see reagents section)</u>
Total 20.5µl

No mixing is required. Continue incubation for 30 more minutes.

Bead cleanup (Ampure XP beads, Beckman Coulter cat#A63881; DynaMag™-96 Side Magnet, Thermo Fisher Scientific cat#12331D)

Let beads adjust to RT for 15-30min.

1) Mix Ampure beads thoroughly, add 25µl beads (1.2:1) to sample (20.5ul). Set pipette to 40µl and pipette x10 in tube. Incubate at RT for 5 min.
2) Place sample on magnet for 5 min until supernatant is clear.
3) Gently remove and discard 44µl of the supernatant without disturbing the beads.
4) With sample still on magnet, add 200µl 80% EtOH, leave for 30s, then gently discard all supernatant. Repeat once for a total of two washings.
5) Quickly spin down tube, put back on magnet and remove all remaining EtOH. (Optionally, if not using a spindown, let beads air dry for 3 min.) Do not let beads over-dry.
6) Resuspend sample in 13µl water, incubate at RT for 2 min.
7) Place sample on magnet for 3-5 min until supernatant is clear.
8) Transfer 12µl of clear supernatant to a new PCR tube (preferably containing library PCR amplification reagents, see next step below).

Library PCR amplification

Thaw reagents at RT and then keep on ice. Reagents can be combined in a new tube during the Ampure cleanup. Then add supernatant directly into tube with reagents.

12µl sample (from previous step)
0.5µl PR2+Ad1 primer (10µM, see reagents section)
<u>12.5µl 2X Phusion HF PCR Master mix (NEB cat#M0531)</u>
Total 25µl

Pipette or flick the tube/strip, quickly spin down. Incubate sample in a PCR machine using the PCRAMP program.

PCRAMP		
Lid 105°C		
95°C	2 min	
94°C	10 sec	16 cycles
60°C	30 sec	
72°C	1 min	
4°C	hold	

Post amplification bead cleanup (Ampure XP beads, Beckman Coulter cat#A63881, DynaMag™-96 Side Magnet Thermo Fisher Scientific cat#12331D)

Let beads adjust to RT for 15-30min.

1) Mix Ampure beads thoroughly, add 23µl beads (0.9:1) to sample (25ul), Set pipette to 40µl and pipette x10 in tube. Incubate at RT for 5 min.
2) Place sample on magnet for 5 min until supernatant is clear.
3) Gently remove and discard 46µl of the supernatant without disturbing the beads.
4) With sample still on magnet, add 200µl 80% EtOH, leave for 30s, then gently discard all supernatant. Repeat once for a total of two washings.
5) Quickly spin down tube, put back on magnet and remove all remaining EtOH. (Optionally, if not using a spindown, let beads air dry for 3 min.) Do not let beads over-dry.
6) Resuspend sample in 13µl water, incubate at RT for 2 min.
7) Place sample on magnet for 3-5 min until supernatant is clear.
8) Transfer 11-12µl of clear supernatant to a new PCR tube.

Quantify and quality check libraries with Qubit (Qubit™ dsDNA HS Assay Kit, Thermo Fisher Scientific cat# Q32851) and TapeStation (TapeStation DNA Screen tape HSD1000, Agilent cat#5067-5584).
Store samples at -20°C until sequencing.[4]

4. Paired-end 41sequencing using a Nextseq500/550 75 cycle or Nextseq2000 P3 50 cycle kit is an affordable option compatible with the insert size of the library.

Reagents; kits/commercial

Single cell lysis kit Invitrogen (Thermo Fisher Scientific cat# 4458235)
NEBNext Ultra™ II RNA First Strand Synthesis Module (New England Biolabs cat#E7771)
NEBNext Ultra™ II Directional RNA Second Strand Synthesis Module (New England Biolabs cat# E7550)
QIAseq FastSelect -rRNA HMR kit (Qiagen cat# 334386)
Agencourt Ampure XP beads (Beckman Coulter cat#A63881)
2X Phusion High-Fidelity PCR Master Mix with HF Buffer (New England Biolabs cat#M0531)
DynaMag™-96 Side Magnet (Thermo Fisher Scientific cat#12331D)
Qubit™ dsDNA HS Assay Kit (Thermo Fisher Scientific cat# Q32851)
TapeStation DNA Screen tape HSD1000 (Agilent cat#5067-5584)

Reagents; home-made or home-mixed

5X Tn5 Tagmentation buffer
Total volume=2ml for 200 samples, aliquot in 4x500µl.

50mM Tris acetate pH7.6	100µl (from 1M stock)
25mM Mg acetate	50µl (from 1M stock)
50% dimethylformamide	1000µl (from 100% stock, add DMFA in a hood)
H2O	850µl

Tn5 enzyme containing only i5 adapters
We have either used home-made or commercially available Tn5, loaded with only the i5 adapter. The home-made Tn5 (kindly provided by Prof. Richard Sandberg) was diluted to 15µM in Tn5 storage buffer before use.

Alternatively, unloaded tagmentase (Tn5 transposase, Diagenode cat#C01070010) was loaded with i5 adapters. Adapter annealing and loading was performed following the manufacturer's protocol (https://www.diagenode.com/files/protocols/PRO-Transposome-Assembly-V2.pdf) but using Tn5MErev annealed to i5MEadapt (see below) and adding twice the amount of this annealed product into the Tn5 adapter loading reaction. Before use, the Tn5 was diluted 1:4, 1:8 or 1:16 in Tn5 storage buffer with similar results.[5]

Tn5 i5 adapters

Tn5MErev: 5'-[phos]**CTGTCTCTTATACACATCT**-3' annealed to:
i5MEadapt: 5'-TCGTCGGCAGCGTC**AGATGTGTATAAGAGACAG**-3'

Tn5 storage buffer

50 mM HEPES, pH 7.2	5 ml 1M
100 mM NaCl	2 ml 5 M
0.1 mM EDTA	20 µl 500 mM
1 mM DTT	0.1 ml 1M
0.1% triton X-100	1 ml 10%
50% glycerol	50ml 100%
H2O	42.8ml

Tagmentation Stop Solution, 1% SDS in water.

5. Likely the Tn5 can be further diluted without impacting the results.

Gap fill buffer

Total volume=1ml for 250 samples, filter, and aliquot in 4x250µl.

165mM tris acetate pH7.5	165µl (from 1M stock RT)
330mM K acetate	330µl (from 1M stock RT)
50mM Mg acetate	50µl (from 1M stock RT)
2.5mM DTT	25µl (from 100mM stock -20°)
1.25mM dATP	12.5µl (from 100mM stock -20°)
1.25mM dCTP	12.5µl (from 100mM stock -20°)
1.25mM dGTP	12.5µl (from 100mM stock -20°)
1.25mM dTTP	12.5µl (from 100mM stock -20°)
1mM beta-NAD	26.5µl (from 37.7mM stock: 25mg+ 1ml H2O -20°)
H2O	353.4µl

Replacement index i7 oligo (Ad2.index)

Replacement oligos (custom made by IDT, ordered in 100nmole scale) diluted to 40µM working concentration in water. (* indicates phosphothioate bonds between the last 8 bases.)

Ad2.index 5'-[phos]CTGTCTCTTATACACATCTCCGAGCCCACGAGAC<u>NNNNNNNN</u>ATCTCGTATGCCGTCT T*C*T*G*C*T*T*G

Ad2.1	TAAGGCGA
Ad2.2	CGTACTAG
Ad2.3	AGGCAGAA
Ad2.4	TCCTGAGC
Ad2.5	GGACTCCT
Ad2.6	TAGGCATG
Ad2.7	CTCTCTAC
Ad2.8	CAGAGAGG
Ad2.9	GCTACGCT
Ad2.10	CGAGGCTG
Ad2.11	AAGAGGCA
Ad2.12	GTAGAGGA

Ad2.13	GTCGTGAT
Ad2.14	ACCACTGT
Ad2.15	TGGATCTG
Ad2.16	CCGTTTGT
Ad2.17	TGCTGGGT
Ad2.18	GAGGGGTT
Ad2.19	AGGTTGGG
Ad2.20	GTGTGGTG
Ad2.21	TGGGTTTC
Ad2.22	TGGTCACA
Ad2.23	TTGACCCT
Ad2.24	CCACTCCT

Gap fill and ligation enzyme mix

Sulfolobus DNA Polymerase IV 100 units (2U/µl) 50µl NEB (Bionordika cat# M0327S)
E. coli DNA Ligase 1000U (10U/µl) 100µl NEB (Bionordika cat# M0205L)

Mix 10µl of polymerase with 20µl of ligase (Unit ratio 1:10), store at -20°C.

PR2+Ad1 primer mix

10µl of Primer 2 (PR2, 100µM stock in water), 10µl Adapter 1 (Ad1, 100µl stock in water) and 80µl water was mixed to make the 10µM PR2+Ad1 primer mix working solution.

Primer 2 / Pr2	CAAGCAGAAGACGGCATACGA
i5 completion primer	AATGATACGGCGACCACCGAGATCTACACTCGTCGGCAGCGTC
